# *Drosophila* glucome screening identifies Ck1alpha as a regulator of mammalian glucose metabolism

**DOI:** 10.1038/ncomms8102

**Published:** 2015-05-21

**Authors:** Rupali Ugrankar, Eric Berglund, Fatih Akdemir, Christopher Tran, Min Soo Kim, Jungsik Noh, Rebekka Schneider, Benjamin Ebert, Jonathan M. Graff

**Affiliations:** 1 Department of Developmental Biology, University of Texas Southwestern Medical Center, Dallas, Texas 75390, USA; 2 Advanced Imaging Research Center, University of Texas Southwestern Medical Center, Dallas, Texas 75390, USA; 3 Department of Pharmacology, University of Texas Southwestern Medical Center, Dallas, Texas 75390, USA; 4 University of Texas, Dallas, Texas 75080, USA; 5 Department of Clinical Sciences, University of Texas Southwestern Medical Center, Dallas, Texas 75390, USA; 6 Division of Hematology, Department of Medicine, Brigham and Women's Hospital, Harvard Medical School, Boston, Massachusetts 02115, USA; 7 Department of Molecular Biology, University of Texas Southwestern Medical Center, Dallas, Texas 75390, USA

## Abstract

Circulating carbohydrates are an essential energy source, perturbations in which are pathognomonic of various diseases, diabetes being the most prevalent. Yet many of the genes underlying diabetes and its characteristic hyperglycaemia remain elusive. Here we use physiological and genetic interrogations in *D. melanogaster* to uncover the ‘glucome', the complete set of genes involved in glucose regulation in flies. Partial genomic screens of ∼1,000 genes yield ∼160 hyperglycaemia ‘flyabetes' candidates that we classify using fat body- and muscle-specific knockdown and biochemical assays. The results highlight the minor glucose fraction as a physiological indicator of metabolism in *Drosophila*. The hits uncovered in our screen may have conserved functions in mammalian glucose homeostasis, as heterozygous and homozygous mutants of *Ck1alpha* in the murine adipose lineage, develop diabetes. Our findings demonstrate that glucose has a role in fly biology and that genetic screenings carried out in flies may increase our understanding of mammalian pathophysiology.

## Abstract (web summary)

Diabetes is associated with aberrations in glucose metabolism. Here the authors perform a genomic screen in fruit flies to identify new regulators of fly glucose metabolism, and show that mice lacking the murine homologue of one of their hits, the protein kinase CK1alpha, in the adipose lineage develop diabetes.

## Graphical abstract

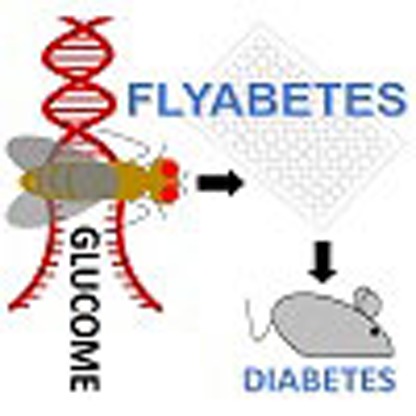

Living cells require a constant supply of energy to maintain integrity and function^1^. In mammals, blood glucose is an essential fuel that on cellular uptake fulfils many of the energy requirements of cells^1^,^2^. A variety of factors, insulin signals notable among them, control the levels of circulating and intracellular glucose^1^,^3^,^4^. Dietary carbohydrates acutely increase blood glucose levels, which in turn trigger pancreatic beta cells to secrete insulin. Insulin then stimulates increased intracellular glucose absorption in muscle, liver and adipose tissues^5^,^6^,^7^. However, the range of homeostatic mechanisms that regulate blood glucose is not yet understood and the genes and pathways altered in obesity and type 2 diabetes are principally unknown^8^,^9^. Therefore, elucidating the ‘Glucome', the complete set of genes involved in glucose regulation, may improve the understanding of the molecular underpinnings of glucose homeostasis, including those disrupted in diabetes, and thereby uncover new diagnostic and therapeutic strategies^9^,^10^.

Key pathways that control metabolism are evolutionarily conserved across a number of species including *Drosophila melanogaster*^11^. For example, ablating insulin-producing cells generates diabetic flies^12^. Moreover, wild-type larvae reared on a high-sugar diet become obese, insulin resistant, and have increased haemolymph (fly blood) glucose and trehalose^13^. For the past decade, *Drosophila* has been fast gaining popularity as a key model system for functional evaluation of mammalian metabolic genes^11^,^14^,^15^. Efforts towards screening for conserved genes that influence carbohydrate homeostasis are already underway, for example, those regulating insulin secretion and dietary insulin resistance, and functional characterization of type 2 diabetes candidates from human genome-wide association studies (GWAS)^16^,^17^,^18^. Until recently, the vast majority of studies in *Drosophila* measured total haemolymph carbohydrates; these primarily reflect trehalose, by far the most prevalent haemolymph carbohydrate with concentrations >100 × glucose^19^,^20^. Quantification of glucose is often carried out alongside trehalose. However, less attention has focused on haemolymph glucose *per se*, so changes in glucose and aspects of its physiological contribution remain uncharacterized^11^. In a study by Pasco and Leopold^21^, the glucose, but not trehalose, fraction in larval haemolymph increased in response to a brief and very concentrated sucrose challenge, indicating that this minor metabolite may be dynamically regulated.

To attempt to further probe glucose, we assess haemolymph glucose changes in *Drosophila* larvae in response to several environmental and genetic modifications, and find that circulating glucose, but not trehalose, levels are regulated in various physiological states and in a number of proof-of-concept (fly homologues of known mammalian glucose regulators) mutants. Next, to identify potential ‘Glucome' candidates, we perform a two-tiered *in vivo* screen focused on the fat body, which is thought to subsume functions of the mammalian adipose tissue and liver, and body wall muscles, which are homologous to mammalian skeletal muscles^11^,^22^. Fat- and muscle-specific RNA interference (RNAi) knockdown of about 1,000 genes followed by glucose measurements uncover 161 unique hyperglycaemia hits, including 76 novel candidates, one of which, *Ck1alpha*, is tested for glucose regulatory role in mice; loss of one or two copies of the mammalian Ck1alpha homologue, *CSNK1a1*, in the adipose lineage results in hyperglycaemic mice. Together our data suggest that haemolymph glucose is sensitive to physiological cues and therefore a relevant readout in *Drosophila* models of metabolism, and that elucidation of the fly ‘Glucome' using a glucose screen may provide candidates with potentially conserved functions in mammals.

## Result

### Environmental cues and metabolic mutations cause flyabetes

**Figure 1:**
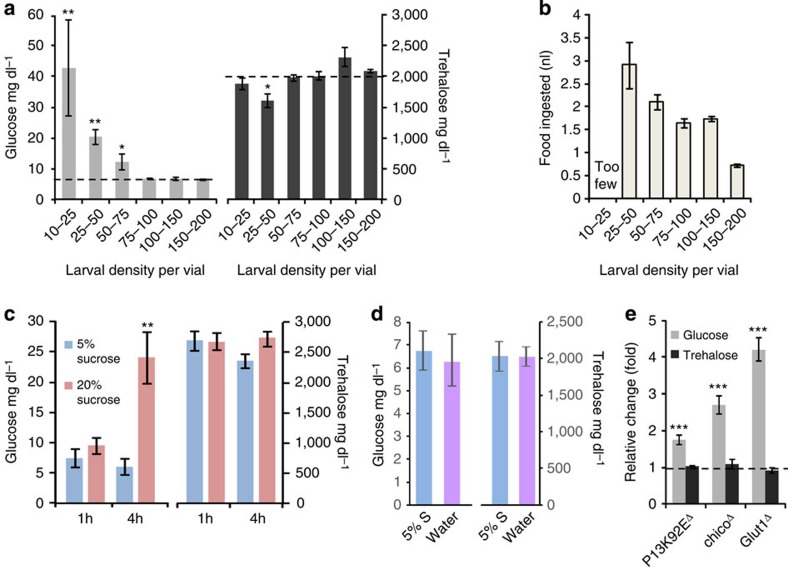
Regulation of fly glucose and trehalose in response to environmental and genetic perturbations. (**a**) Circulating glucose (left) and trehalose (right) levels were measured in mid-third instar *w*^*1118*^ larvae cultured in various density conditions. *n*=4 each (⩾10 larvae per replicate). (**b**) Quantification of feeding rates of *w*^*1118*^ larvae cultured in various density conditions. *n*=3 each (30 larvae per replicate). (**c**) *w*^*1118*^ larvae were provided 5% and 20% sucrose solutions and after 1 or 4 h, glucose (left) and trehalose (right) levels were measured. *n*=4 each (⩾10 larvae per replicate). (**d**) *w*^*1118*^ controls were provided water and 5% sucrose and after 4 h, glucose and trehalose levels were measured. *n*=4 each (⩾10 larvae per replicate). (**e**) Glucose and trehalose levels of P-element mutants for three mammalian glucose regulatory proof-of-principle genes. *n*=4 each (⩾10 larvae per replicate). Error bars indicate s.e.m. Statistical significance was assessed by two-tailed Student's *t*-test, *P<0.05, **P<0.01, ***P<0.001.

To test if haemolymph glucose is responsive to physiological cues in *Drosophila*, we examined glucose levels in several settings in which the mammalian blood glucose levels would vary and found that haemolymph glucose is subject to physiological and environmental regulation. For example, control *w*^*1118*^ mid-third instar larvae reared under low-density conditions (<75 larvae per vial) had significantly higher circulating glucose, up to ∼40 mg dl^−1^, compared with those reared under mid to high density conditions (75–200 larvae per vial), whose levels generally ranged between 6 and 9 mg dl^−1^ in our assay. Notably, trehalose levels (>1,500 mg dl^−1^) were not altered by density (Fig. 1a). We reasoned that uncrowded rearing might decrease competition and increase food availability and consumption—a notion supported by quantitative feeding measurements taken at varying larval densities (Fig. 1b). To assess the possibility that food might affect glucose levels, we reared *w*^*1118*^ larvae in standard conditions (5% sugars, mid-high density), transferred them to 5 or 20% sucrose solutions and allowed them to feed for 1 or 4 h. After the acute conditioning, we measured haemolymph glucose and trehalose levels and found that 4 h of 20% sucrose significantly increased glucose, but not trehalose, levels (Fig. 1c). These data are consistent with a previous report identifying a trend of elevated glucose levels but unchanged trehalose levels in response to a short high-sucrose challenge, in which larvae were prefasted and then fed a concentrated 120% sucrose solution for 1 h (ref. 21). Thus, fluctuations in circulating glucose due to transient metabolic challenges might be a general phenomenon. A study by Geminard *et al*.^23^ reported that total haemolymph carbohydrates was increased in third instar larvae following 24 h of starvation on low-sucrose media. To test if acute sucrose deprivation produced changes in glucose and/or trehalose levels, we placed *w*^*1118*^ larvae in water, alongside controls in 5% sucrose. *w*^*1118*^ larvae fasted in water for 4 h showed unchanged haemolymph carbohydrates relative to those consuming 5% sucrose, indicating that caloric deprivation, at least in the short term, does not increase glycaemia (Fig. 1d).

To determine whether the circulating glucose and/or trehalose is responsive to loss-of-function of key metabolic genes, we measured both fractions in mutant larvae with P-element insertions in *Drosophila* homologues of proof-of-concept genes: *chico* (fly *IRS*, insulin signalling), *PI3K* (insulin signalling) and *Glut1* (glucose transporter). We found that only glucose was significantly increased in these ‘flyabetes' mutants (Fig. 1e). Together these data suggest that glucose may reflect mammalian biology, and therefore, may have potential relevance to mammalian blood glucose.

### Homologous function of known glucose effectors

**Figure 2:**
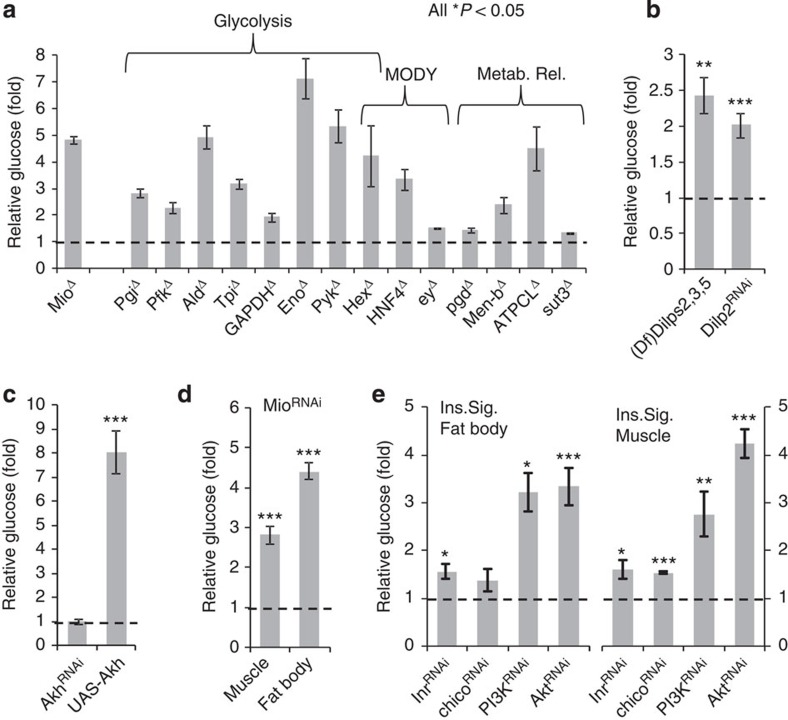
Homologous function of key proof-of-principle glucose regulatory genes. (**a**) Glucose levels were measured in quadruplicate for the indicated fly proof-of-concept mutants and relative levels compared with controls were plotted (⩾10 larvae per replicate). (**b**,**c**) Relative glucose levels of larvae with altered expression of insulin (**b**) and glucagon (*Akh*) (**c**). *n*=4 each (⩾10 larvae per replicate). (**d**) Relative glucose levels, compared with controls, of muscle *(Mef2-Gal4)* and fat body *(Dcg-Gal4)*-specific *Mio* knockdown. *n*=4 each (⩾10 larvae per replicate). (**e**) Glucose levels of flies with fat body or muscle RNAi of various insulin signalling components. *n*=4 each (⩾10 larvae per replicate). Error bars indicate s.e.m.. Statistical significance was assessed by two-tailed Student's *t*-test, *P<0.05, **P<0.01, ***P<0.001. Ins. Sig., insulin signalling.

To further probe the significance of glucose in *Drosophila*, we measured the glucose levels in larvae with disruptions in various other metabolic genes. We first evaluated a mutant with a P-element inserted at the *Mio* locus, the fly homologue of mammalian ChREBP, a glucose-responsive transcription factor that regulates a battery of key glucose metabolic pathways^24^. Ubiquitous loss of function of *Mio* has been reported to increase haemolymph carbohydrates in *Drosophila* larvae^25^; we also found that glucose levels were significantly elevated in *Mio* mutants (Fig. 2a). Since glycolytic pathway genes are regulated by Mio, we tested glucose levels in several glycolysis mutants; these larvae also showed significant elevations in circulating glucose (Fig. 2a). One of the glycolysis genes, *Hex* (hexokinase), is the causative mutation underlying a subset of patients with MODY (maturity onset diabetes of the young), an uncommon monogenic form of diabetes^26^. So we obtained the two available fly mutants of MODY candidates: *HNF4* (*HNF4A*) and *ey* (*Pax4*); all three had ‘flyabetes' (Fig. 2a). We also assessed mutants for genes involved in different aspects of carbohydrate metabolism: *Pgd* (pentose phosphate pathway), *Men-b* (Krebs cycle), *ATPCL* (fatty acid and acetyl-CoA synthesis) and *sut3* (fly sugar transporter). Again, we observed significant increases in glucose levels (Fig. 2a). We next investigated the homologues of key mammalian glucoregulatory hormones, insulin (*Dilp*) and glucagon (*Akh*). We found that a deficiency line that deletes *Dilps2*, *3* and *5* was hyperglycaemic; elevated total carbohydrates (glucose plus trehalose) have also been reported in larvae with ablated *Dilps2*, *3* and *5*-producing brain cells^12^. We also performed RNAi using a ubiquitous *Actin-Gal4* driver for each *Dilp* (*1*–*7)*: only the loss of *Dilp2* moderately increased glucose levels (Fig. 2b). A previous study did not detect glucose changes in response to *Dilp2* knockdown alone^27^; however, glucose measurements were made in late-stage wandering (fasting) larvae. In our studies with continually feeding mid-third instars, it is possible that Dilp2, the most abundantly expressed insulin-like peptide^27^, may play a larger role in regulating carbohydrate homeostasis. Consistent with this notion, a transgene only overexpressing *Dilp2* rescued the metabolic phenotypes of a mutant lacking *Dilps2*, *3* and *5* (ref. 12). We also found that the overexpression of *Akh* produced marked glucose elevations, comparable to the effect of glucagon in increasing blood glucose levels in mammals^28^ (Fig. 2c). These data further support the notion that glucose is an important readout of fly metabolism.

To explore the fat body and muscles as potentially relevant tissues to target for loss-of-function screening using the two-component *UAS/Gal4* system, we induced fat body (*Dcg-Gal4* driver) and muscle (*Mef2-Gal4* driver) knockdown of *Mio*. We found that both the knockdowns produced significant glucose elevations (Fig. 2d), highlighting key functions for both tissues. Next, we examined whether tissue-specific roles of insulin signalling in glucose homeostasis were also conserved in the fly. We found that fat body and muscle-restricted RNAi-induced repression of insulin signalling components increased haemolymph glucose levels (Fig. 2e). These data support the potential that the *UAS/Gal4* system in conjunction with glucose measurements may be an effective screening tool for Glucome candidates, as observed in other studies, and one that could increase the understanding of tissue-specific and tissue-common roles.

### Fat- and muscle-specific screening of kinases

To further evaluate the potential of the fly model to dissect the Glucome, we probed the tissue-specific contribution of kinases by inhibiting their function in fat body and muscle. Kinases are well-established drug targets and are integral components of many metabolic networks, indicating that this pool might be enriched in glucose regulators (‘hits')^29^. We conducted fat body- and muscle-specific knockdown with 95 kinase *UAS-dsRNA* lines—to avoid lethality that might result from ubiquitous or embryonic inhibition, to assess tissue-specific functions, and to compare hit rates and Glucomes between tissues. We quantitated glucose concentrations (mg dl^−1^) in four independent haemolymph samples from RNAi knockdown and control feeding mid-third instar larvae. Genes were designated as hits if RNAi loss-of-function larvae had increased haemolymph glucose levels relative to controls in a statistically significant manner as initially evaluated using a two-tailed Student's *t*-test (*P*<0.05). Following the primary screen, here and throughout the described studies, positives for these criteria were then subjected to the more stringent Wilcoxon rank-sum test, which resulted in overall higher *P* values and confirmed more than 95% of the positives from the *t*-test evaluations. On the basis of the *P* values obtained from the Wilcoxon test, we reclassified the hits as follows—‘high confidence', if Wilcoxon test *P*<0.05 (significant); ‘medium confidence', if Wilcoxon test *P*=0.057 (borderline significant); and ‘low confidence' (not significant), if Wilcoxon test *P*>0.057. All *t*-test Wilcoxon test hits from primary screens (high, medium and low) were further validated by further quadruplicate glucose measurements, a minimum of three times. Each of these validation repetitions was separated in time by at least 1 month, in part, to reduce any false positives due to biological variation. The validation measurements were compared with the primary screen hits using a binomial test with 95% confidence intervals to access whether there were any significant differences between the initial test and the validation data. Individual fold-change comparisons were carried out by evaluating the 95% confidence interval of the primary fold change using the variance from the replicate measurements^30^,^31^.

**Figure 3:**
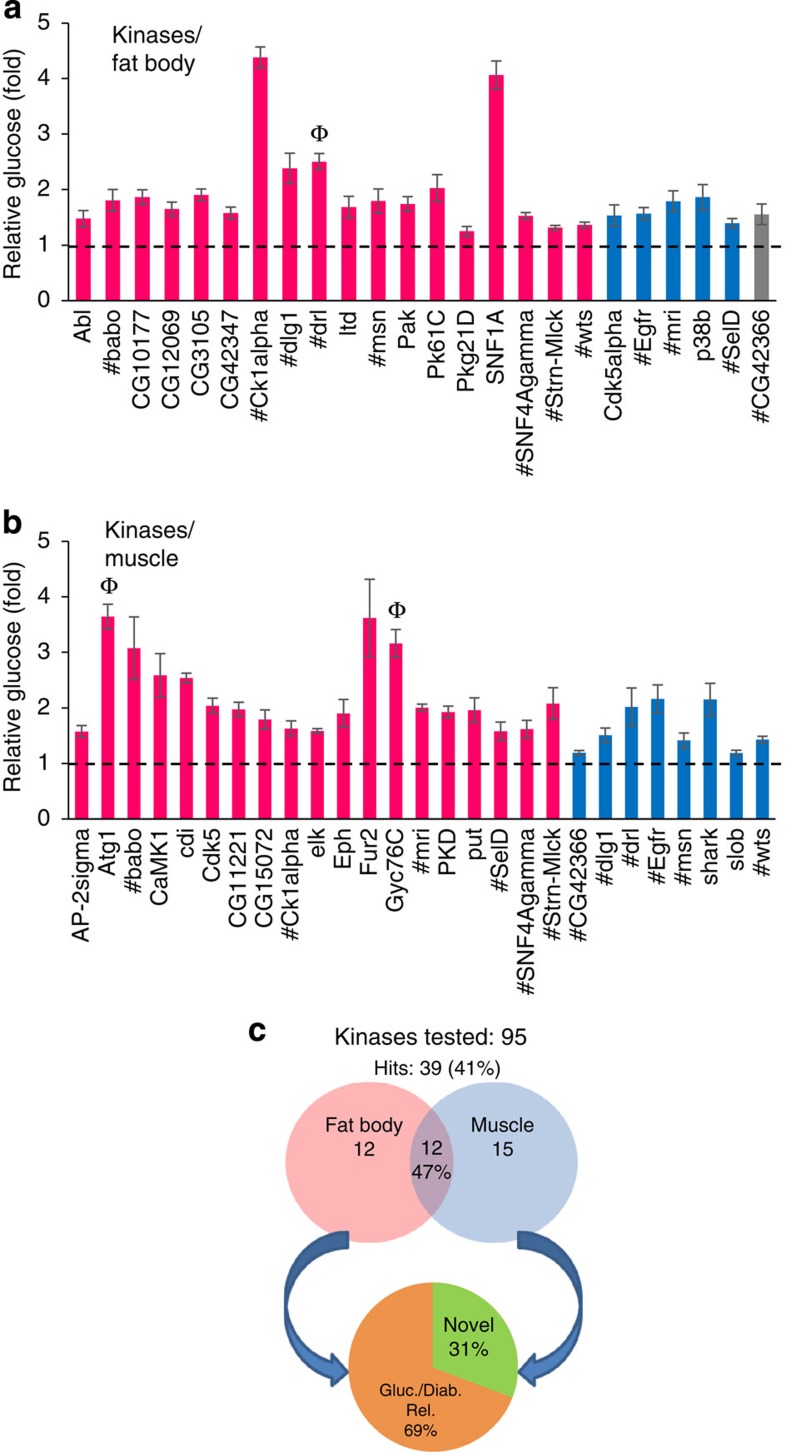
Tissue-specific Glucome RNAi screening of 95 kinases. (**a**,**b**) Primary screening, using quadruplicate independent samples, of 95 kinase genes produced significant glucose elevations for 24 genes in the fat body (**a**) and 27 genes in the muscle (**b**); *n*=4 each (⩾10 larvae per replicate), statistical significance was initially assessed by two-tailed Student's *t*-test (*P*<0.05, error bars indicate s.e.m.). # indicates a common hit in fat body and muscle screens. Student's *t*-test-positive (*P*<0.05) ‘hits' were reclassified based on *P* values from the Wilcoxon rank-sum test. High confidence to left and in magenta: *P*<0.05, medium confidence toward right and in blue: *P*=0.057, and low confidence at right end and in grey: *P*>0.057. Genes are alphabetically listed in each confidence level. After the primary screen, at least three repeat independent glucose measurements, in quadruplicate, were done for each hyperglycaemia candidate identified in the *t*-test/Wilcoxon test evaluations and then assessed by a binomial 95% confidence interval evaluations; Ф symbol above bar indicates the very few genes not validated with at least 95% confidence in the binomial evaluations of the three independent repetitions. (**c**) The proportion of hyperglycaemia kinase hits from primary screen in either tissue, overlapping hits in both tissues, and genes previously associated with glucose metabolism or diabetes and novel genes are indicated.

We screened 95 kinases and detected significant hyperglycaemia in 25% (24 of 95) and 28% (27 of 95) of fat body and muscle knockdowns, respectively (Fig. 3a–c, Supplementary Table 1). We next did an intertissue comparison and found that approximately half of the hits overlapped between fat body and muscle. Together, this identified a total of 39 (41%) unique Glucome candidates. To evaluate functional classes, signalling cascades, mammalian homology and commonality to identified mammalian glucoregulators, we referenced all hits to the databases Flybase, PANTHER, GeneCards and to available literature. For about 69% of kinase candidates, we uncovered at least one reference indicating potential involvement of their mammalian counterpart in glucometabolic pathways or diabetes risk (GWAS). These data support the notion that the fly is suitable for tissue-specific delineation of previously unrecognized hyperglycaemia candidates, potentially conserved in the regulation of mammalian glucose homeostasis.

### Fat- and muscle-specific screening of NHRs

**Figure 4:**
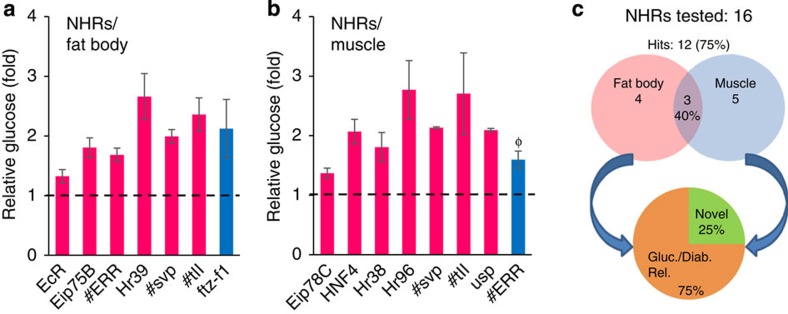
Tissue-specific Glucome RNAi screening of 16 NHRs. (**a**,**b**) Primary screening of 16 NHRs yielded seven hyperglycaemia hits in fat body (**a**) and eight hyperglycaemia hits in muscle (**b**), respectively; *n*=4 each (⩾ 10 larvae per replicate), statistical significance was evaluated as outlined in Fig. 3. In brief, initially assessed by two-tailed Student's *t*-test (*P*<0.05, error bars indicate s.e.m.). # indicates a common hit in fat body and muscle screens. Hits were reclassified based on *P* values from the Wilcoxon rank-sum test, as in Fig. 3. High confidence (in magenta): *P*<0.05, medium confidence (in blue): *P*=0.057, and low confidence (in grey): *P*>0.057. Genes are alphabetically listed in each confidence level. After the primary screen, three repeat glucose fold changes for hyperglycaemia candidates were assessed by a 95% confidence interval; Ф symbol above bar indicates the genes not validated with 95% confidence. (**c**) The proportion of primary screen hyperglycaemia NHR hits in either tissue, overlapping hits in both tissues, and genes previously associated with glucose metabolism or diabetes, and novel genes are indicated.

The nuclear hormone receptor (NHR) subclass of transcription factors plays critical roles in energy homeostasis, and several *Drosophila* NHRs have links to carbohydrate metabolism (*ERR*, *Hr38* and *Hr96*)^22^,^32^,^33^. We tested RNAi lines for the available 16 of 18 total fly NHRs in larval fat body and muscle. We detected hyperglycaemia in 75% (12 of 16) of these lines, with roughly equal hit rates in fat body (44%, 7 of 16) and muscle (50%, 8 of 16), respectively (Fig. 4a–c, Supplementary Table 2). Similar to what we observed with the kinases, a high percentage of hits (40%) overlapped between the two tissues and 75% had links to glucose regulation or diabetes. Novel genes comprised 3 of the 12 candidates.

### Fat- and muscle-specific screening of transcription factors

**Figure 5:**
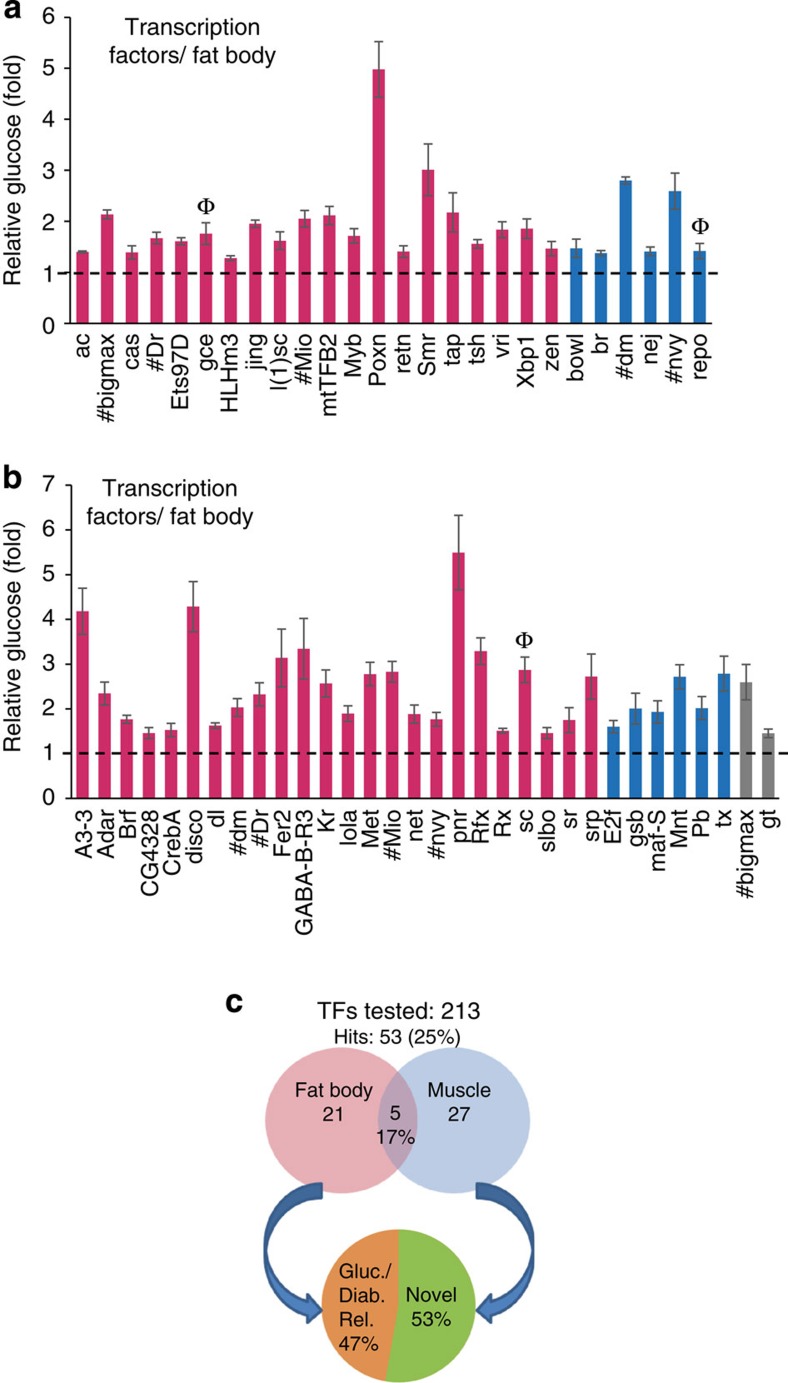
Tissue-specific Glucome RNAi screening of 213 non-NHR class transcription factors. (**a**,**b**) Transcription factors (213) tested in the primary screen, loss of 26 genes in the fat body (**a**) and 32 genes in the muscle (**b**) significantly increased haemolymph glucose; *n*=4 each (⩾10 larvae per replicate), statistical significance was evaluated as outlined in Fig. 3. Initially assessed by two-tailed Student's *t*-test (*P*<0.05, error bars indicate s.e.m.). # indicates a common hit in fat body and muscle screens. Hits were reclassified based on *P* values from the Wilcoxon rank-sum test, as in Fig. 3. High confidence (in magenta): *P*<0.05, medium confidence (in blue): *P*=0.057, and low confidence (in grey): *P*>0.057. Genes are alphabetically listed in each confidence level. After the primary screen, three repeat glucose fold changes for hyperglycaemia candidates were assessed by a 95% confidence interval; Ф symbol above bar indicates the genes not validated with 95% confidence. (**c**) The proportion of hyperglycaemia transcription factor hits in either tissue, overlapping hits in both tissues and genes previously associated with glucose metabolism or diabetes, and novel genes are indicated.

We next screened non-NHR transcription factors because transcription factors are key regulators that can facilitate the elucidation of downstream effectors, and they have fewer known links to metabolism than kinases and NHRs. We screened glucose levels that resulted from the knockdown of 213 transcription factor *UAS-dsRNA* lines in fat body and muscle. Similar to what we observed with the kinases and NHRs, the two tissues appeared to have a relatively similar sized Glucome; 12% (26 of 213) and 15% (32 of 213) of transcription factors were hits in fat body and muscle, respectively (Fig. 5a–c, Supplementary Table 3). However, other features of the transcription factor glucose data were distinct—the overall hit rate 25% (53 of 213) was significantly lower than in the kinase and NHR subsets, as was the common pool (17%). 47% of hits were previously implicated in potential Glucome functions. Notably, the proportion of genes (53%) not linked to glucose regulation was significantly higher than in the other two pools indicating that they may be involved in glucose-responsive target gene expression.

### Fat body-specific screening of randomly selected genes

**Figure 6:**
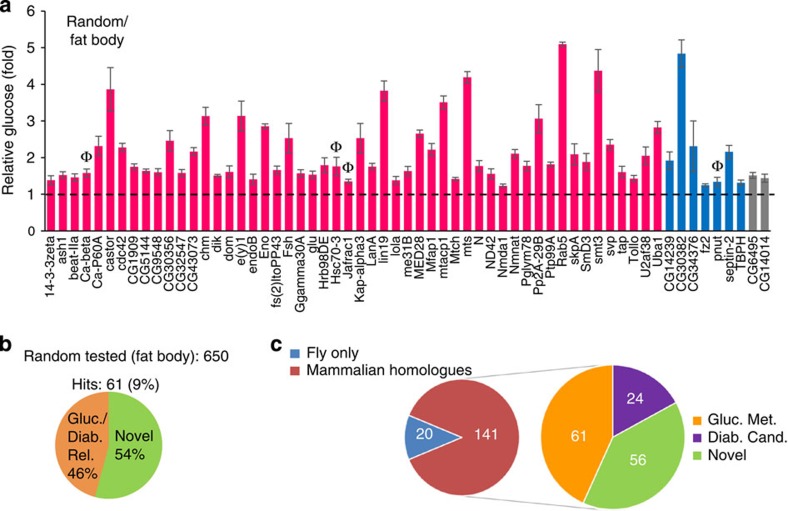
Fat body-specific Glucome RNAi screening of ∼650 randomly selected genes. (**a**) Approximately 650 genes were examined in the fat body, 61 whose loss in the fat body resulted in significant hyperglycaemia; statistical significance was evaluated as outlined in Fig. 3. *n*=4 each (⩾10 larvae per replicate). Statistics were initially assessed by two-tailed Student's *t*-test (*P*<0.05, error bars indicate s.e.m.). Hits were reclassified based on *P* values from the Wilcoxon rank-sum test. High confidence (in magenta): *P*<0.05, medium confidence (in blue): *P*=0.057 and low confidence (in grey): *P*>0.057. Genes are alphabetically listed in each confidence level. After the primary screen, three repeat glucose fold changes for hyperglycaemia candidates were assessed by a 95% confidence interval; Ф symbol above bar indicates the genes not validated with 95% confidence. (**b**) Number of primary screen hits linked to glucose regulation and novel hits are indicated. (**c**) Classification of candidates obtained in all RNAi-glucose screens. Diab. cand., diabetes candidate;Gluc. met., glucose metabolism.

We next expanded to a larger and randomly selected pool of genes, comprising all biological groups, in part, to attempt to delineate the overall percentage of genes that contribute to the Glucome. We focused on the fat body, and found that 61 of about 650 (∼9%) random RNAi transgenes resulted in significant glucose elevations (Fig. 6a,b, Supplementary Table 4). Although this percentage was much lower than the kinases and NHRs, it was on the order of what we observed in the fat body transcription factor screen (12%). Slightly more than half of random hits (54%) had no known relation to glucose metabolism, again similar to the transcription factor screen. So it seems plausible that some of these new candidates may also have conserved homeostatic functions in mammalian glucose homeostasis.

A summary of all hyperglycaemia hits obtained from the various fat body- and muscle-targeted RNAi screening is represented in Fig. 6c.

### Haemolymph glucose and trehalose do not track together

**Figure 7:**
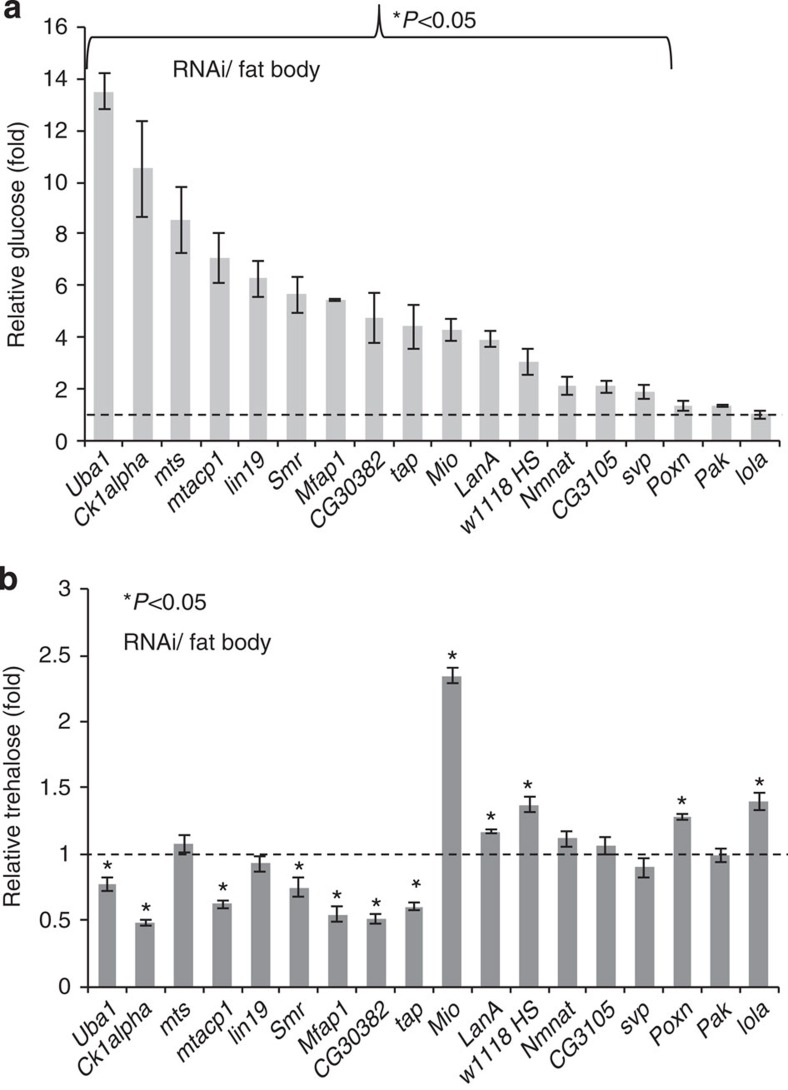
Haemolymph glucose and trehalose fractions correlations. (**a**,**b**) Glucose (**a**) and trehalose (**b**) levels were measured in 17 fat body hyperglycaemia candidates, high-sucrose fed *w*^*1118*^, and control larvae; dotted line indicates control concentrations. Error bars indicate s.e.m.. Statistical significance was assessed by two-tailed Student's *t*-test, *P<0.05, **P<0.01, ***P<0.001.

To further assess a potential association between glucose and trehalose fractions in haemolymph, we assayed 17 hyperglycaemia ‘flyabetes' candidates from the fat body RNAi screen, high-sugar-reared *w*^*1118*^ and control larvae concurrently for glucose and trehalose. We found that glucose elevations in the haemolymph did not necessarily correspond with trehalose elevations, as only four out of 18 candidates tested were both hyperglycaemic and hypertrehalosemic. Among these are high-sugar-reared *w*^*1118*^ larvae and *Mio*^*RNAi*^, consistent with previously published studies^13^,^24^. Remarkably, seven out of 18 hyperglycaemia hits, including *Uba1*^*RNAi*^ and *Ck1alpha*^*RNAi*^ that displayed the largest glucose fold changes, had significantly lower trehalose levels. 5 of 18 candidates showed glucose elevations but unchanged trehalose levels, while three others (*Poxn*, *Pak* and *lola*) were not significantly hyperglycaemic in this instance but *lola*^*RNAi*^ had elevated trehalose (Fig. 7a,b). We also noted that with the exception of *Mio*, even significant trehalose changes were small, not exceeding or falling below 50% of controls while glucose levels varied up to ∼15 times controls. Therefore, glucose had a much greater dynamic range than trehalose, and did not appear to track with trehalose.

### *CSNK1a1* loss in adipose lineage produces hyperglycaemic mice

**Figure 8:**
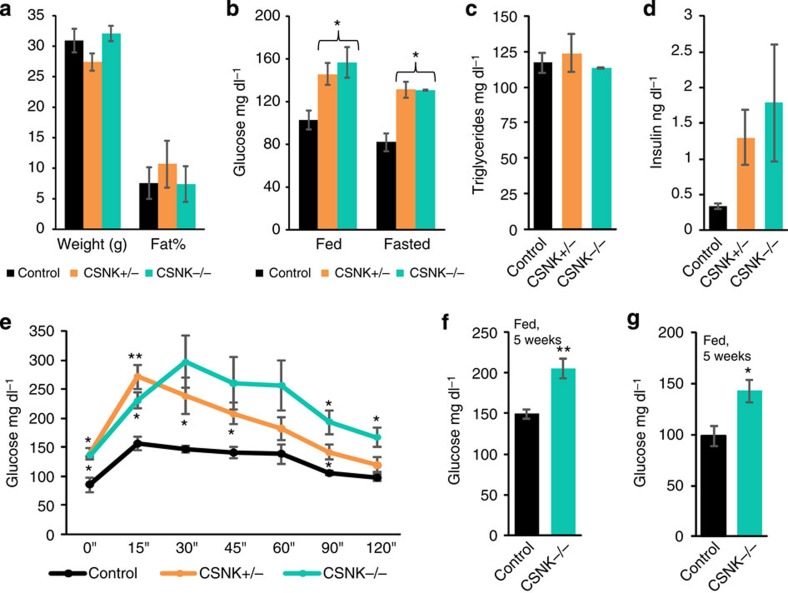
Loss-of-function of fly *Ck1alpha* homologue (*CSNK1a1*) in mouse adipose tissue progenitors induces hyperglycaemia. (**a**) Plots show weights and per cent fat content (NMR spectrometry) of 3.5-month-old control and mutant mice fed standard chow. *n*=4 each. (**b**) Fed and fasted glucose was measured in control, and homozygous and heterozygous *PPARγ-CSNK1a1* knockouts. *n*=4 each. (**c**,**d**) Fasted plasma triglycerides (**c**), and insulin levels (**d**) detected in control and mutant mice. *n*=3 each. (**e**) GTT was performed on 3.5-month-old control and mutant mice. *n*=4 each. (**f**,**g**) Blood glucose was measured in 5-week (**f**) and 10-week-(**g**) old control and *PPARγ-CSNK1a1* mutant mice. *n*=6 each. Error bars indicate s.e.m.. Statistical significance was assessed by two-tailed Student's *t*-test, *P<0.05, **P<0.01. Data shown for male mice; phenotype was less pronounced in females.

*Ck1alpha* emerged as a strong novel hyperglycaemia candidate in our RNAi-glucose screen; loss of *Ck1alpha* in both fat body and muscle of third instar larvae produced significant haemolymph glucose elevations 100% of the time. To investigate if the murine homologue of *Ck1alpha* (*CSNK1a1*) has a conserved role in mammalian metabolism, we combined one or two *CSNK1a1*^*f/f*^ conditional ‘floxed' alleles with a *PPARγ-tTA* ‘knockin' allele^34^,^35^ and *TRE-Cre* (*PPARγ-tTA; TRE-Cre; CSNK1a1*^*f/+*^, and *PPARγ-tTA; TRE-Cre; CSNK1a1*^*f/f*^) to delete *CSNK1a1* throughout the mammalian adipose lineage. All mice were maintained on a regular chow diet after weaning. We then analysed body mass, fat content (using NMR) and glucose levels (fed and fasted) at 3.5 months of age. We found no significant difference in body weight or fat content between heterozygous or homozygous *CSNK1a1* mutant and control (*PPARγ-tTA; TRE-C*re or *PPARγ-tTA; CSNK1a1*^*f/f or f/+ or +/+*^) siblings at 3.5 months (Fig. 8a). Notably, we did detect significant hyperglycaemia in both homozygous and heterozygous mutants, using a standard finger stick blood glucose monitor that is widely used for human patients (Fig. 8b). We also measured triglycerides and insulin in plasma, derived from 3.5-month-old mice, in a clinical laboratory setting; mutants had normal triglycerides, but insulin levels trended higher hinting at possible insulin resistance (Fig. 8c,d). We then performed a glucose tolerance test (GTT) and found that heterozygous and homozygous mutants displayed significantly impaired glucose tolerance, and the homozygous mutants appeared to have a trend towards more severe disease; GTT area under the curve for heterozygous and homozygous mutants, as compared with controls, were 146% and 179%, respectively (Fig. 8e). We also examined homozygous mutants and control siblings at 5 weeks of age, soon after weaning, and at 10 weeks of age; both times we detected significantly elevated (fed) glucose in mutants (Fig. 8f,g); thus, these mice seem to develop diabetes at a young age. These data indicate that the deletion of one or two copies of *CSNK1a1* in adipose tissues does not alter body or fat mass, but does produce significant hyperglycaemia. Thus, *Ck1alpha/CSNK1a1* appears to regulate glucose metabolism in flies and mammals.

## Discussion

Mammals maintain glucose in an optimal range and disruptions in glucose homeostasis can lead to type 1 and 2 diabetes^8^,^36^. Therefore, identifying the ‘Glucome' may be invaluable to developing effective clinical strategies. In *Drosophila melanogaster*, trehalose, rather than glucose, predominates in haemolymph^11^,^19^,^20^. Recent studies quantify both trehalose and glucose concentrations in fly haemolymph, and several studies indicate the importance of fly glucose^13^,^16^,^17^,^18^,^21^. To attempt to extend these observations, we developed and optimized a colorimetric glucose assay that was direct, faster and cheaper than conventional trehalose assays. We then attempted to unravel the role of glucose using several physiological and genetic tests. The results are consistent with the notion that in *Drosophila* glucose is a relevant circulating carbohydrate, that *Drosophila* glucose biology could be predictive for human glucose homeostasis, and that flies may be a key testing ground to uncover aspects of the Glucome.

We examined the regulation of fly glucose in several physiological states. In our assay, we found that haemolymph glucose is typically maintained within 6–9 mg dl^−1^ in control *w*^*1118*^ mid-third instar *Drosophila* larvae. Further, larvae cultured in low-density conditions ate more and had higher glucose levels. Hyperglycaemia was also induced by high-sucrose meals; yet the trehalose levels were unchanged by the culture conditions or the caloric challenges; a similar trend was reported by Pasco and Leopold^21^. These data support the notion that circulating glucose is sensitive to environmental cues, while in these settings the vastly dominant trehalose does not appear to be. This idea appeared to be born out in proof-of-concept mutants as flies with mutations in insulin signalling components or the glucose transporter displayed high glucose, but normal trehalose, levels. Notably, the dynamic range was substantially greater for glucose than for trehalose. Together, our studies, and reports of others^13^,^16^,^17^,^18^, highlight the potential of glucose measurements in fly metabolic biology.

We then tested a series of fly mutants of genes known to affect the blood glucose levels in mammals. Mutations in the fly homologue of *ChREBP* (*Mio*) have been reported to increase the glucose levels in larvae^25^. We found that the alteration of Mio and additional proof-of-concept candidates, such as glycolysis genes, MODY homologues, insulin *(Dilp)* and glucagon (*Akh*), all produced hyperglycaemia in a manner predicted from mammalian studies. We also explored the possibility that the fly might serve as a model of tissue-specific glucose regulation, a critical aspect of mammalian physiology. We performed fat body- and muscle-restricted RNAi-mediated knockdown of *Mio* and insulin signalling components; the resultant mutants were hyperglycaemic. This indicates that these fly tissues, like their mammalian counterparts, appear to be critically involved in maintaining euglycaemia. Taken together, the data support the possibility that glucose might be a relevant haemolymph carbohydrate in flies and one pertinent to mammalian metabolism.

We next explored the possibility that glucose quantitation might serve as a screening tool to extend our knowledge of the ‘Glucome', those genes that control systemic carbohydrate levels; contributions by other labs to the ‘Glucome' include identification and characterization of genes that control insulin secretion, insulin sensitivity, and human diabetes candidates^16^,^17^,^18^. For this, we combined *in vivo* fat body- and muscle-specific RNAi in conjunction with a 96-well format glucose assay, potentially suited to high throughput screening. We initially evaluated an identical set of kinases, NHRs, and transcription factors in both tissues. Primary screen hits, done in quadruplicate, were selected by Student's *t*-test, classified by Wilcoxon rank-sum test, and at least three replicate quadruplicate validation measurements were analysed by 95% confidence intervals. We detected hyperglycaemia after RNAi knockdown in both tissues, of ∼40% (39 of 95) of kinases, 75% (12 of 16) of NHRs, and 25% (53 of 213) of transcription factors. The higher ‘hit' rate observed for kinases is consistent with their functions within general signalling cascades, while transcriptions factors may execute more tissue-specific functions. Further support for this notion derives from the observation that a larger number of kinases (47%) than transcription factors (17%) were overlapping hits in both tissues. A roughly equal percentage produced hyperglycaemia from either tissue—for kinases 25% (24 of 95) and 28% (27 of 95), and for transcription factors 12% (26 of 213) and 15% (32 of 213), in fat body and muscle, respectively. These data are consistent with the possibility of a relatively conserved Glucome size and equivalent contributions by both tissues in glucose regulation. Approximately 30% of kinase and 53% of transcription factor candidates were novel in glucose regulation. Since a large percentage of hits of either class were, in at least one report, previously linked to glucose homeostasis or diabetes (GWAS), it seems plausible that some of these new candidates will also regulate mammalian blood glucose.

We also performed fat body-only screening of a larger group of randomly selected genes comprising a host of functional classes. The fat body hit rate (9%) for this group was significantly lower than for the kinases (25%) but close to that for transcription factors (12%). Since the percentage of kinase (25%, 28%) and transcription factor (12%, 15%) candidates was roughly the same between fat body and muscle, the percentage derived from the unbiased fat body (9%) screen may hint at the relative proportion of glucoregulatory factors in the genome. About half of the random hits were not associated with mammalian glucose homeostasis. If this is the general trend, then a significant proportion of the Glucome remains undiscovered.

Glucose and trehalose are both important metabolites in insects^13^,^16^,^17^,^18^,^21^ and yet in our assay conditions, we found that fly glucose is responsive to several physiological and genetic changes known to alter blood mammalian glucose levels. Further, in our studies, haemolymph glucose and trehalose levels only infrequently tracked together; for example, loss of *Mio* causes both glucose and trehalose elevations. Our data show that changes in glucose and trehalose often do not correspond; for example, loss of *Ck1alpha* and *Uba1* causes high glucose levels and low trehalose levels. Therefore, *Drosophila* glucose physiology may mirror attributes of mammalian glucose homeostasis, and it may be useful to measure fly glucose in attempts to elucidate aspects of the ‘Glucome' that are relevant to mammalian glucose dynamics.

One reason we interrogated fly glucose homeostasis was to attempt to uncover genes that may have mammalian glucoregulatory function or that may be human diabetes genes. To explore the possibility of conserved function notion, we generated mice in which one of the strong flyabetes hits, *Ck1alpha* (*CSNK1a1*), was deleted in the murine adipose lineage. In part, we selected *Ck1alpha* because it was a kinase, a class of genes that are often druggable targets^29^; and small-molecule inhibitors of CK1 have been reported^37^. The results appeared to validate the overall strategy; mice with loss of *CSNK1a1* in murine adipose tissue were hyperglycaemic and glucose intolerant. We intentionally maintained the mice on normal (low-fat) chow, rather than the more provocative high-fat setting. While both *CSNK1a1* homozygous and heterozygous knockouts had similar levels of fed glucose and fasted glucose, when they were challenged with a GTT, homozygous knockouts had more severely impaired glucose tolerance than heterozygous knockouts. Fasted insulin levels also trended higher in homozygous relative to heterozygous knockouts. Such haploinsufficiency can have advantages in clinical studies^38^, for example, requiring lower doses and potentially reducing the risk of side effects. Although the mutant mice had diabetes, the fat content and triglycerides appeared normal indicating that the effect might be more directly related to glucose homeostasis *per se*, rather than an obesogenic effect. This supports the potential that the hyperglycaemia results from non-autonomous signals. Such adipokines are widely sought out, and molecular interrogation of the CK1 models, for example, using RNA-Seq, mass spectroscopy or other methods, may help identify candidate molecules. These data further highlight potential metabolic similarities between flies and mammals. This supports the possibility that the various tests and screening methods developed here might be useful tools to identify novel regulators of glucose homeostasis, to characterize underlying mechanisms and glucoregulatory functions, and to reveal total and tissue-specific Glucome size.

In summary, we analysed the role of *D. melanogaster* circulating carbohydrates in various physiological states and through *in vivo* genetic screens. Consistent with other studies^13^,^15^,^16^,^17^,^18^,^21^, our data highlight glucose as a relevant circulating carbohydrate in flies, identify many new factors of hyperglycaemia and highlight key roles of tissue-specific glucose regulation. From the various RNAi screens, we obtained a total of 161 unique hits, of which 141 have identified mammalian homologues. Among these, 56 are novel hyperglycaemia candidates (Fig. 6c). Of the 161 hits with Student's *t*-test *P*<0.05, 76% (123) were high confidence hits with Wilcoxon test *P*<0.05, 21% (33) were medium confidence hits with Wilcoxon test *P*=0.057, and 3% (5) were low confidence hits with Wilcoxon test *P*>0.057, in the fat body or muscle screens. Ninety-three per cent (150) of all candidates were found to be confidently validated with 95% confidence. Loss of function of one of these candidates, *Ck1alpha*, in the mouse adipose lineage resulted in significant blood glucose elevations; it seems likely that other novel hits from these, and additional more exhaustive fly screens, may also display metabolic regulatory roles in mammals. Identifying the complete ‘Glucome' may lead to a more comprehensive understanding of glucose homeostasis and the hereditary predictors of diabetes. Hence, characterization of promising Glucome candidates in mammalian glucose homeostasis may present potential preventive or therapeutic targets in type 2 diabetes and related metabolic disorders.

## Methods

### Fly strains and culture conditions

Flies were reared on standard cornmeal–molasses–yeast–agar food. All *UAS-dsRNA*, *Actin-Gal4* and *Mef2-Gal4* drivers and mutant lines were obtained from the Bloomington Stock Center. *Dcg-Gal4* flies were described^39^. High-sugar food was prepared by adding sucrose (15 g 100 ml^−1^) to standard food. RNAi loss-of-function crosses and other stocks were maintained at 25 °C. The common laboratory strain *w*^*1118*^ was used as the control line. In RNAi screening, and other experiments involving RNAi, progeny from the crosses between paternal *w*^*1118*^ and maternal *Gal4* driver served as controls, to maintain uniformity in all tissue-specific screening.

### Larval collection

After egg laying, larvae were cultured in medium-to-high-density conditions (75–150 larvae per vial, unless otherwise indicated) for 6–7 days before harvesting feeding mid-third instars. Vials were rid of older wandering larvae on the sides and food surface. Larvae were collected in 630-μm mesh-fitted baskets (Genesee) and washed to get rid of adherent food particles. Baskets were lowered into a tray of water to allow dead flies to float out, and then into a shallow tray of 2 M NaCl so that larvae float to the top. Larvae were transferred with a paintbrush to Pyrex 9 depression glass spot plates (Corning), and used for different assays.

### Glucose and trehalose assays

Larvae were divided into four piles (10–12 larvae each) on a strip of parafilm. Larvae were bled by tearing the cuticle with Dumont 5 forceps (Electron Microscopy Sciences). Two μl of colourless haemolymph was aspirated from each pile and separately transferred to 96-well plates (Thermo-Scientific) containing 0.1% *N*-Phenylthiourea (Sigma-Aldrich) in 50 μl PBS. Autokit Glucose reagent (150 μl, Wako) was added to each well, and incubated at room temperature for 20 min before measuring absorbance at 505 nm. Glucose concentration was calculated from a standard curve generated with the manufacturer's glucose standards. For trehalose assays, 8 μl of dilute haemolymph was treated with 5 μl of (diluted 8 × ) porcine kidney trehalase (Sigma) overnight at 37 °C. Treated sample (10 μl) was assayed for trehalose as described for glucose.

### Food ingestion quantification

Larvae fed Coomassie dye-supplemented media were harvested, washed in water and methanol, blotted dry and transferred to 1.5 ml Eppendorf tubes on ice (30 larvae per sample, in triplicate). Larvae were homogenized in 200 μl of 100% methanol. Samples were centrifuged at 14,000 r.p.m. for 10 min; 175 μl of the supernatant was transferred to fresh tubes containing 175 μl of dH_2_O and centrifuged again. Absorbance of this supernatant was measured at 595 nm, and dye amount (food ingested) was calculated by comparing with a standard curve generated from a range of Coomassie dye concentrations.

### Mouse studies

The *PPARγ-tTA* and *TRE-Cre* mice were maintained as described and used to generate controls and *CSNK1a1* loss-of-function mutants. *CSNK1a1* conditional knockout mice (loxP sites flank exon 3) were provided by Benjamin Ebert, Harvard University. Mice were fed normal chow (4% fat, Teklad). Sample size was chosen as needed for statistical significance by a two-tailed Student's *t*-test. Control and mutant male mice were analysed for glucose levels at 5 weeks, 10 weeks and 3.5 months of age. Fed blood glucose was measured at the end of the light cycle, between 17:00 and 18:00 hours, using a standard glucometer (Contour). For GTTs, 1.25 mg glucose per 1 g mouse weight was injected intraperitoneally after a 5–6-h daytime fast; blood glucose levels were measured at the indicated times. Fat content was measured using a minispec mq10 NMR Analyzer (Bruker) at the UT Southwestern Medical Center Mouse Metabolic Phenotyping Core (MMPC). Necropsies were performed on male mice ⩾3.5 months of age. Plasma triglyceride and hormone tests were also done at the UTSW MMPC. Veterinary care was provided by the Division of Comparative Medicine. Animals were maintained under the UT Southwestern Medical Center Animal Care and Use Committee guidelines according to current NIH guidelines under animal protocol 2010-0015.

## Additional information

**How to cite this article:** Ugrankar, R. *et al*. *Drosophila* glucome screening identifies Ck1alpha as a regulator of mammalian glucose metabolism. *Nat. Commun.* 6:7102 doi: 10.1038/ncomms8102 (2015).

## Supplementary Material

Supplementary Information Supplementary Tables 1-4
