# Supplementary material for: Drosophila glucome screening identifies Ck1alpha as a regulator of mammalian glucose metabolism
Source: Nat Commun. 2015 May 21;6:7102. doi: 10.1038/ncomms8102 (PMC4455130; doi:10.1038/ncomms8102)
Supplement: Supplementary Information — Supplementary Tables 1-4 [file ncomms8102-s1.pdf]

Supplementary Information

Supplementary Table1

Kinase/Fat body

Fly gene	Mamm. homolog	Wilcoxon P-value
**Glucose metab. Related genes**		
CG3105	Pask	0.028571
CG10177	p90RSK	0.028571
CG12069	Pka-like	0.028571
**dlg1**	Dlg1/2/3/4	0.028571
Pak	Pak1/2/3	0.028571
Pk61C	PDK1	0.028571
SNF1A	Prkaa1/2	0.028571
**SNF4Aɣ**	Prkag1/2/3	0.028571
**Strn-Mlck**	Titin/Mlck	0.028571
Cdk5alpha	Cdk5r2	0.057143
**Egfr**	Egfr	0.057143
**mri**	Btbd10	0.057143
p38b	Mapk11/13	0.057143
**Genes related to Diab. incidence**		
**babo**	TGFBR1	0.029401
**msn**	Mink1	0.028571
Pkg21D	Prkg1/2	0.028571
**wts**	Lats1/2	0.028571
**Novel genes in glucose metab.**		
Abl	Abl1/2	0.028571
**Ck1alpha**	Csnk1a1	0.028571
CG42347	Mylk2/3/4	0.028571
**drl**	Ryk	0.028571
ltd	n/a	0.028571
**SelD**	Sephd1/2	0.057143
**CG42366**	Ick/Mak	0.114286

Muscle

Fly gene	Mamm. homolog	Wilcoxon P-value
**Glucose metab. Related genes**		
Cdk5	Cdk5	0.028571
CG15072	Sik1/2/3	0.028571
Eph	EphA/B	0.028571
**mri**	Btbd10	0.028571
**SNF4Aɣ**	Prkag1/2/3	0.028571
**Strn-Mlck**	Titin/Mlck	0.028571
CaMKI	CaMKI	0.028571
Fur2	Furin	0.028571
PKD	Prkd3	0.028571
**dlg1**	Dlg1/2/3/4	0.057143
**Egfr**	Egfr	0.057143
slob	Pxk	0.057143
**Genes related to Diab. incidence**		
AP-2sigma	Ap2s1	0.028571
**babo**	TGFBR1	0.028571
Gyc76C	Gucy2c	0.028571
put	TGFBR2	0.028571
**msn**	Mink1	0.057143
**wts**	Lats1/2	0.057143
**Novel genes in glucose metab.**		
Atg1	Ulk1/2	0.028571
cdi	tesk1	0.028571
CG11221	Sbk1	0.028571
**Ck1alpha**	Csnk1a1	0.028571
elk	Kcnh3/4/8	0.028571
**SelD**	Sephd1/2	0.028571
**CG42366**	Ick/Mak	0.057143
**drl**	Ryk	0.057143
shark	zap70	0.057143

Supplementary Table1: Kinase candidates from RNAi screening in fat body and muscle

(a)Kinase hits in fat body (left) and muscle (right) screens, and known mammalian homologs are listed, with P-values for the Wilcoxon rank sum test. High confidence: P < 0.05, Medium confidence: P = 0.057, Low confidence: P > 0.057. Genes are alphabetically listed in each confidence level. **BOLD** = common hits in fat body and muscle.

Supplementary Table2

NHRs/Fat body

Fly gene	Mamm. homolog	Wilcoxon P-value
**Glucose metab. Related genes**		
EcR	Nr1h2/3/4	0.028571
Hr39	LRH1-like	0.028571
**svp**	COUP-TF	0.028571
**Genes related to Diab. incidence**		
**ERR**	ESRRa/b	0.028571
**Novel genes in glucose metab.**		
Eip75B	n/a	0.028571
**tll**	n/a	0.028571
ftz-f1	Nr5a1/2	0.057143

Muscle

Fly gene	Mamm. homolog	Wilcoxon P-value
**Glucose metab. Related genes**		
Hr38	Nr4a1/2/3	0.01587
**svp**	COUP-TF	0.028571
usp	RXRa/b/g	0.028571
**Genes related to Diab. incidence**		
Eip78C	Nr1D1/2	0.028571
HNF4	HNF4a	0.028571
Hr96	VDR	0.028571
**ERR**	ESRRa/b	0.057143
**Novel genes in glucose metab.**		
**tll**	n/a	0.028571

Supplementary Table2: Nuclear hormone receptor candidates from RNAi screening in fat body and muscle

List of NHR candidates, their known mammalian homologs, and Wilcoxon test P-values, in the fat body (left) and muscle (right) screens. High confidence: $P < 0.05$, Medium confidence: $P = 0.057$, Low confidence: $P > 0.057$. Genes are alphabetically listed in each confidence level. **BOLD** = common hits in fat body and muscle.

Supplementary Table 3

TF/Fat body

Fly gene	Mamm. homolog	Wilcoxon P-value
**Glucose metab. Related genes**		
**bigmax**	MLXip	0.028571
**Mio**	ChREBP	0.028571
Smr	SMRT	0.028571
**dm**	Myc	0.057143
**Genes related to Diab. incidence**		
ac	Ascl1	0.028571
l(1)sc	Ascl1	0.028571
tap	NeuroD1	0.028571
vri	Nfil3	0.028571
Xbp1	Xbp1	0.028571
nej	Crebbp	0.057143
**Novel genes in glucose metab.**		
cas	Casz1	0.028571
**Dr**	Msx1/2/3	0.028571
Ets97D	Gabpa	0.028571
gce	n/a	0.028571
HLHm3	Hes2	0.049746
jing	Aebp2	0.028571
mtTFB2	TFBM1/2	0.028571
Myb	MYB	0.028571
Poxn	n/a	0.028571
retn	ARID3A	0.028571
tsh	n/a	0.028571
zen	n/a	0.028571
bowl	Osr1	0.057143
br	n/a	0.057143
**nvy**	CBFA2T	0.057143
repo	n/a	0.057143

Muscle

Fly gene	Mamm. homolog	Wilcoxon P-value
**Glucose metab. Related genes**		
A3-3	ATF3	0.028571
CrebA	Creb3L	0.028571
dl	Relb	0.028571
**dm**	Myc	0.028571
lola	ZBTB	0.028571
**Mio**	ChREBP	0.028571
slbo	C/EBP	0.028571
sr	Egr1	0.029401
maf-s	Mafk	0.057143
**bigmax**	MLXip	0.1
gt	TEF	0.1
**Genes related to Diab. incidence**		
Adar	Adar	0.028571
CG4328	Lmx1a/b	0.049746
GABA-B-R3	Gabbr1/2	0.028571
Kr	KLFs	0.028571
Rfx	Rfx1/2/3	0.028571
E2f	E2f	0.057143
gsb	Pax	0.057143
**Novel genes in glucose metab.**		
Brf	Brf1	0.028571
disco	n/a	0.028571
**Dr**	Msx1/2/3	0.028571
Fer2	Bhlhf42	0.028571
Met	n/a	0.028571
**nvy**	CBFA2T	0.028571
net	n/a	0.028571
pnr	GATA4/5	0.028571
Rx	Rax	0.028571
sc	Ash1	0.028571
srp	GATA	0.029401
taxi	n/a	0.028571
Mnt	Mnt	0.057143
Pb	Hoxa2/b2	0.057143

Supplementary Table 3:Transcription factor candidates from RNAi screening in fat body and muscle

List of non-NHR transcription factor candidates, their known mammalian homologs, and Wilcoxon test P-values, in the fat body (left) and muscle (right) screens.High confidence: $P < 0.05$, Medium confidence: $P = 0.057$, Low confidence: $P > 0.057$. Genes are alphabetically listed in each confidence level. **BOLD** = common hits in fat body and muscle.

Supplementary Table 4

Random/Fat body

Fly gene	Mamm. homolog	Wilcoxon P-value
**Glucose metab. Related genes**		
14-3-3zeta	Ywhaz/ Sfn	0.028571
Cdc42	cdc42	0.028571
Eno	Eno1/2/3	0.028571
Gγ30A	gng13	0.028571
Kap-alpha3	Kpna	0.028571
lola	ZBTB	0.028571
mts	Ppp4C	0.028571
Rab5	Rab5a/b/c	0.028571
svp	COUP-TF	0.028571
Pglym78	Pgam1/2	0.028571
pnut	Sept1/2/4/5	0.057143
**Genes related to Diab. incidence**		
ash1	Ash1l	0.029401
Ca-beta	Cacnb	0.028571
Ca-P60A	SERCA1	0.028571
endoB	Sh3glb1/2	0.028571
glu	Smc4	0.028571
Jafrac1	PRDX1	0.028571
LanA	Lama3/5	0.028571
lin19	cul1	0.029401
N	Notch1/2/3	0.028571
Pp2A-29B	Ppp2r1a/b	0.028571
skpA	skp1a	0.028571
tap	Neurog1	0.028571
Tollo	IL1	0.028571
CG30382	Psma6	0.057143
CG34376	n/a	0.057143
fz2	Fzd1/6/7	0.057143
CG6495	LRP11	0.059072

Fly gene	Mamm. homolog	Wilcoxon P-value
**Novel genes in glucose metab.**		
beat-IIa	n/a	0.028571
cas	Casz1	0.028571
CG1909	Rapsn	0.028571
CG5144	Ckb/m/mt	0.028571
CG9548	n/a	0.028571
CG30356	n/a	0.028571
CG32547	n/a	0.028571
CG43073	RIMBP3	0.028571
chm	Kat6b/8	0.028571
dik	Tada3	0.028571
dom	Srcap	0.028571
e(y)1	taf9/9b	0.028571
f2(2)ltoPP43	n/a	0.028571
Fsh	FSHR	0.028571
Hrb98DE	n/a	0.028571
Hsc70-3	Hspa5/ BiP	0.028571
me31B	Ddx2	0.028571
MED28	MED28	0.028571
Mfap1	Mfap1a/b	0.028571
mtacp1	Ndufab1	0.028571
Mtch	Mtch1/2	0.028571
ND42	Ndufa10	0.028571
Nmda1	Grin1	0.028571
Nmnat	Nmnat1/2/3	0.028571
Ptp99A	Ptprg	0.028571
SmD3	Snrpd3	0.028571
smt3	SUMO1/2/3	0.028571
U2af38	U2af1	0.028571
Uba1	Uba1	0.028571
CG14239	n/a	0.057143
septin-2	sept6/10/11	0.057143
TBPH	Tardbp	0.057143
CG14014	n/a	0.11021

Supplementary Table 4:Random candidates from RNAi screening in fat body

61 random hyperglycemia candidates identified in fat body, mammalian homologs, and P-values for the Wilcoxon rank sum test. High confidence: $P < 0.05$, Medium confidence: $P = 0.057$, Low confidence: $P > 0.057$.Genes are alphabetically listed in each confidence level.
